# Mini-FLOTAC and Kato-Katz: helminth eggs watching on the shore of lake Victoria

**DOI:** 10.1186/1756-3305-6-220

**Published:** 2013-07-31

**Authors:** Beatrice Barda, Henry Zepherine, Laura Rinaldi, Giuseppe Cringoli, Roberto Burioni, Massimo Clementi, Marco Albonico

**Affiliations:** 1 Laboratory of Microbiology San Raffaele Hospital, Milan, Italy; 2 Department of Veterinary Medicine and Animal Productions, Section of Veterinary Parasitology and Parasitic Diseases, University of Naples Federico II, Naples, Italy; 3 Bukumbi Hospital, Mwanza, Tanzania; 4 Ivo de Carneri Foundation, Milan, Italy

**Keywords:** Soil-transmitted helminths, Mini-FLOTAC technique, Kato-Katz method, Lake Victoria Tanzania

## Abstract

**Background:**

One of the challenges for monitoring helminth control programmes based on preventive chemotherapy is the lack of a copro-parasitological gold–standard method that combines good sensitivity with quantitative performance, low cost, and easy-to-learn technique.

The aim of our study was to evaluate and compare, the WHO recommended quantitative diagnostic technique (Kato-Katz) and the Mini-FLOTAC.

**Methods:**

Mini-FLOTAC is an innovative method based on floatation of helminths eggs with two different solutions (FS2 and FS7) using a close system (Fill-FLOTAC) with 5% fixative. Kato-Katz was performed following WHO recommendation. The study was carried out in a rural part of Tanzania, close to Lake Victoria, where the laboratory facilities are fairly scarce, and the basic technique used in the local laboratory (direct smear) was taken as reference standard.

**Results:**

201 children were screened for intestinal helminths and 91% of them were found to be positive. The agreement among the three techniques was calculated with k Cohen coefficient and was fairly good (k = 0.4), although the Mini-FLOTAC results were more sensitive for hookworm (98%) with FS2, and for *S.mansoni* (90%) with FS7 followed by Kato-Katz (91% and 60% respectively) and direct smear (30% and 10% respectively)*.* A good agreement was found between Mini-FLOTAC and Kato-Katz (k = 0.81) with FS7 (k = 0.76) for hookworm diagnosis and a fairly good one for *S.mansoni* diagnosis (k = 0.5). For both infections we had a poor agreement between the two quantitative techniques and the direct smear (k<0.3). Kato-Katz diagnosed a higher number of eggs (calculated by arithmetic mean) both for hookworm (455 vs 424 EPG) and for *S.mansoni* (71 vs 58 EPG) compared with the Mini-FLOTAC, but the differences were not significant (p = 0.4).

**Conclusions:**

Mini-FLOTAC is a promising technique, comparable and as sensitive as the Kato-Katz, which is the recommended method in intestinal helminthology for monitoring helminth control programmes. A comparative advantage of the Mini-FLOTAC is that it comprises of a closed system with preserved samples that both protects the operators and allows subsequent examination of the samples. Further studies are needed to validate the mini-FLOTAC with other quantitative techniques (McMaster) and in different settings where other soil-transmitted helminths are also endemic.

## Background

Almost two billion people are infected worldwide by soil-transmitted helminths (STH) (*Ascaris lumbricoides, Trichuris   trichiura,  Ancylostoma   duodenale/Necator americanus*) and schistosomes (*Schistosoma mansoni*, *S. haematobium* and *S. japonicum*) [1]. These infections are under the umbrella of neglected tropical diseases (NTD) whose control is advocated by the WHO and other International Agencies with the strategy based on preventive chemotherapy [2]. Among the 112 countries considered in need of preventive chemotherapy, 42 are African; all of the East African countries, and specifically Tanzania, is an area where these infections are widespread [3-5]. In 2010 global data on drug coverage for STH registered that 38% of pre-school children, 26% of school-children and 30% of the total number of children in need of treatment have actually been treated in the African regions [1]. In particular, schistosomes are currently reported in 52 countries which all need preventive chemotherapy and more than 70% of all cases of schistosomiasis live in African countries [1].

Among a few, two challenges are on the frontline for STH control programmes. The first one is assessing the impact of interventions on prevalence and intensity of infections; the second one is monitoring drug efficacy. Both require standard quantitative techniques, and presently WHO is developing guidelines for recommending the most appropriate methods. As previously mentioned, the areas most affected by these infections are developing countries, where laboratories and health care facilities are frequently scarcely equipped; this may contribute to impairment of the control programmes both on mapping and on monitoring the impact of the intervention. An ideal diagnostic technique to be applied in field laboratories of STH endemic countries should be able to combine robustness, simplicity, low cost and good sensitivity. Kato Katz is the quantitative technique recommended for the diagnosis of STH [6]. Other techniques commonly used for faecal egg count (FEC) of helminth parasites are adapted from veterinary to human parasitology, such as McMaster [7] and FLOTAC [8].

More recently, molecular tools such as PCR and multiplex PCR have been improved, next to the direct techniques, as qualitative diagnostic methods for parasitic infections in laboratories where the equipment is available, although this is not the case for rural and district laboratories in developing countries [9,10].

The mini-FLOTAC is a new simplified diagnostic device recently developed and simplified from FLOTAC techniques that, despite high sensitivity for the diagnosis of intestinal parasitic infections [11,12], are limited by their complexity and need of a special centrifuge [13]. Mini-FLOTAC is a low cost method, which does not require any expensive equipment or energy source, so it can be comfortably used in developing countries. The aim of this study was to compare Kato-Katz with mini-FLOTAC techniques for the diagnosis of intestinal helminth infections in a setting where resources are scarce. The main purpose was to focus on the affordability of this new and innovative technique and its transferability into peripheral laboratory, in order to improve diagnosis and facilitate parasitological monitoring of STH/NTD control programmes.

## Methods

### Study site

Mwanza district is an area in north-western Tanzania, which lies on the shore of Lake Victoria; it comprises of many small towns and villages where the main population’s income comes from farming and fishing. Kigongo and Isamilo, the study sites, are two villages within this area; the former is placed directly on the lake and the latter is 2 miles away from the lake. Soil conditions, poor quality of drinking water, and constant contact with contaminated fresh water are hazards for the transmission of intestinal parasitic infections. The closest main hospital is Bukumbi hospital, where approximately 300 000 people are referred for medical care. This parasitological survey together with transfer of knowledge and techniques has been carried out as part of the collaboration programme between AISPO NGO (Italian association for solidarity among people), linked with San Raffaele hospital, and Bukumbi hospital in July 2012.

In each village there is a primary school and our survey was conducted in school children, selected at random in each class. One faecal sample was collected and analyzed for intestinal parasitic infections within the same day.

### Parasitological methods

The stool samples were collected and analyzed within 24 hours with three different techniques: direct smear, Kato-Katz and mini–FLOTAC method. Approximately two mg of stool were used to perform the direct smear. The Kato-Katz was performed using the 41.7 mg template, according to the WHO recommendation [6]. Mini-FLOTAC stands on two components, the base and the reading disc; the base includes two 1-ml flotation chambers, which are designed for optimal examination of fecal sample suspensions (total volume = 2 ml): which permits a maximum magnification of ×400. The stools were processed as follows for the mini-FLOTAC technique (analytic sensitivity = 10 eggs/larvae/cysts per gram of faeces). Five grams of stool were weighed and placed in the fill-FLOTAC, a plastic device part of the kit that facilitates filtration, dilution and homogenization of the sample, diluted with 5 ml of 5% formalin and thoroughly homogenized and filtered. Two ml of the suspension (1 gram of stool + 1 ml of formalin) were directly added to 18 ml of each of the two floating solutions, namely FS2 (saturated sodium chloride; density = 1.20) and FS7 (zinc sulphate; density = 1.35). The floatation solutions are the same as those described in the FLOTAC protocols [8]. Two Mini-FLOTACs were performed for each sample, one filled with the faecal suspension in FS2 and the other with the faecal suspension in FS7. Before reading the slide and translating the reading dish, an average time of 10 minutes was needed for the eggs to float. Eggs of intestinal helminths were detected and counted within the grid.

### Statistical analysis

Results were entered into an Excel file. Analyses were performed using the Epidat programme (version 3.1; Area of Health Analysis and Information Systems Pan American Health Organization, January 2006); the results were analysed by 2x2 contingency tables and K Cohen was calculated to assess the agreement among all the three diagnostic techniques. Kappa (k) statistic was employed to determine the strength of agreement using the following criteria: ≤ 0 = poor, 0.01-0.20 = slight, 0.21-0.40 = fair, 0.41-0.60 = moderate, 0.61-0.80 = substantial and 0.81-1 = almost perfect. Mean eggs per gram (EPG) of faeces were calculated by the arithmetic mean and difference between means of EPG was compared using the student-t test. We checked for any significant difference by calculating interference about proportions (p) in two independent populations with Graphpad prism 6.0, the level of significance was set at p value <0.05 and the confidence interval (CI) was calculated at a level of 95%.

### Ethical issues

The study was reviewed and approved by the Ethics Committee of the Faculty of Medicine, San Raffaele Hospital, Milan, Italy. A separate ethical clearance was obtained from the Bukumbi hospital management board. All children were given an informed consent form to be read and approved by their parents/guardian before being enrolled into the study. After the survey, all school children were treated with praziquantel and albendazole according to the WHO recommendations for preventive chemotherapy [2]. Data was stored kept anonymously and patients were identified by code; the study data were safely filed and stored in a cabinet within the data management unit of the research site and remained confidential.

## Results

Two hundred and one children were enrolled in the survey, 89 (44%) males, aged between 4 and 19 years old (mean 11 years). One hundred and eighty two (91%) children were found positive for any helminthic infection.

**Figure 1:**
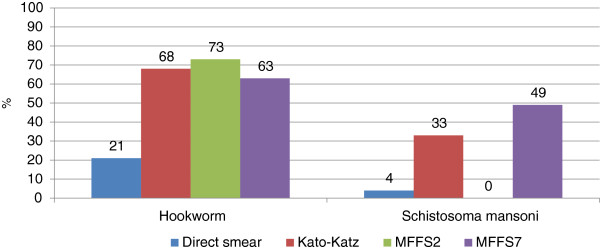
Prevalence of intestinal helminth infections with the three techniques.

**Figure 2:**
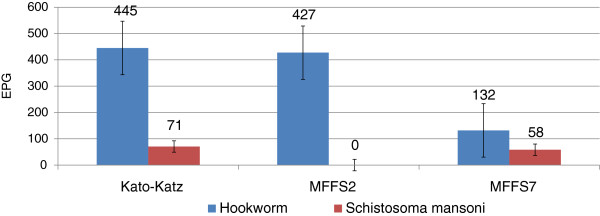
Eggs per Gram of faeces (arithmetic mean± standard error) with Kato Katz and Mini-FLOTAC methods.

Results of the parasitological survey are shown in Figure 1; 150 children (75%) were found positive for hookworm, 106 (53%) were positive for *S. mansoni* and 14 (7%) were positive for other parasitic infections, such as *Strongyloides stercoralis, Enterobius vermicularis* and *T. trichiura* using any of the three techniques. Among the three techniques the agreement (k Cohen coefficient) that resulted was fairly good (k = 0.4); the highest number of diagnosis of hookworm (73%) and *S.mansoni* (49%) that were detected with the mini-FLOTAC FS2 and FS7, respectively, followed by Kato-Katz (68% and 33% respectively) and the direct smear (21% and 4% respectively). K Cohen among techniques was calculated (Table 1) and a good agreement was found for hookworm between Kato-Katz and mini-FLOTAC both FS2 (k = 0.81) and FS7 (k = 0.76) and between mini-FLOTAC FS2 and FS7 (k = 0.78), whereas a low agreement was registered with direct smear (k< 0.3). For *S.mansoni* agreement between Kato-Katz and mini-FLOTAC, FS7 was fairly good (k = 0.5), but very low with direct smear (k< 0.2). As Kato-Katz and mini-FLOTAC are quantitative techniques, we calculated the difference between arithmetic means detected for hookworm and *S.mansoni* (Figure 2). For hookworm the highest egg count was diagnosed with Kato-Katz (455 mean EPG) followed by mini-FLOTAC FS2 (427 mean EPG) and FS7 (132 mean EPG) and a statistically significant difference was found between Kato-Katz and mini-FLOTAC FS7 (p < 0.001) and between mini-FLOTAC FS2 and FS7 (p < 0.001), but no difference was noticed between Kato-Katz and mini-FLOTAC FS2 (p < 0.68). As for *S.mansoni* the highest count was detected with Kato-Katz (71 mean EPG) followed by mini-FLOTAC FS7 (58 mean EPG) although they were not statistically different (p < 0.4). FS2 did not detect any *S. mansoni* eggs.

**Table 1:** K Cohen calculated for agreement among techniques and t- student for comparison of arithmetic means

	**k Cohen**	**CI 95%**	**p value**	**Mean of differences (CI 95%)**
**Hookworm**				
DS vs KK vs MFFS2 vs MFFS7	0.42	0.34-0.5	NA	NA
DS vs KK	0.22	0.15-0.29	NA	NA
DS vs MFFS2	0.18	0.11-0.24	NA	NA
DS vs MFFS7	0.30	0.2-0.40	NA	NA
KK vs MFFS7	0.76	0.67-0.86	< 0.0001	- 313.5 (−437.7;-189.3)
KK vs MFFS2	0.81	0.72-0.90	0.68	- 17.86 (−103.1; 67.40)
MFFS2 vs MFFS7	0.78	0.68-0.87	< 0.0001	- 295.6 (−402; -189. 3)
***Schistosoma mansoni***				
DS vs KK vs MFFS7	0.22	0.13-0.30	NA	NA
DS vs KK	0.18	0.07-0.28	NA	NA
DS vs MFFS 7	0.09	0.03-0.15	NA	NA
KK vs MFFS7	0.53	0.42-0.64	0.3558	- 12.38 ( −38.75;14)

*DS:* direct smear.

*KK:* Kato-Katz.

*MFFS2-7:* Mini-FLOTAC floatation solution 2–7.

*NA:* not applicable.

## Discussion

Studies on drug efficacy and egg reduction rate have identified the need for a low-cost, sensitive, accurate and easy-to-perform quantitative test, that could be used in developing countries where intestinal helminth infections are endemic [14]. So far such an ideal method does not exist and some authors have claimed that the choice of a single diagnostic method limits the diagnostic accuracy and have suggested matching more than one technique and combining results to achieve a more comprehensive diagnosis [15]. Kato-Katz has been the recommended method for a long time, but lately studies on comparison among techniques have proved that it is not the most sensitive and accurate method for eggs counts [16,17], although easy to perform and affordable [18,19]. The McMaster method is commonly used in veterinary medicine and it has lately been used for a multicentre evaluation of anthelminthic drug efficacy also in human parasitology with good results [16]. In this and other studies [17] McMaster has shown a good accuracy and sensitivity making it worthy of consideration as a valid and low cost alternative to Kato-Katz technique. Mini–FLOTAC was created with the idea of matching a good sensitivity with a low cost technique; we compared this new borne technique with the Kato-Katz thick smear for the first time and results are encouraging, as the two techniques showed similar sensitivity and egg count accuracy. This finding is important as the Kato-Katz is the recommended technique for STH diagnosis in public health, but mini-FLOTAC detected a higher number of positive children for hookworm (73% vs 68%) as well as for *S.mansoni* (49% vs 33%). It is also important to observe that the prevalence of the other helminths was too low to be taken as a meaningful comparison and further studies are needed. As for egg counts, both methods detected a similar mean of eggs per gram, a bit higher with the Kato-Katz both for hookworm (455 vs 427 with mini-FLOTAC) and *S.mansoni* (71 vs 58 with mini-FLOTAC), but were not statistically different. It is worth noticing the great difference of performance between the two floatation solutions; in fact the saturated saline (FS2) detects more hookworm than the zinc sulphate solution (FS7) not only in sensitivity but also in egg count (427 vs 132), on the other hand, saturated saline (FS2) does not detect *S. mansoni*.

Additional criteria of the “ideal” diagnostic method are easy-to-learn and quick to perform and are low-cost. The mini-FLOTAC technique could be carried out correctly by local laboratory technicians after a short period of training. Moreover, we compared the timing to perform Kato-Katz and mini-FLOTAC and actually the latter was quicker, as it needed 3 min for sample preparation, 10 min for the eggs to float and approximately 5 min for the reading, whereas Kato-Katz needed 1-2 min for the sample preparation, 30 min for the glycerol to clarify the eggs and 3–5 min for reading. As for the costs, the Kato-Katz kits are purchased at a low price for developing countries and the mini-FLOTAC is under negotiation with WHO to be donated free of charge, most devices of both tests are reusable, and no further equipment is needed. Regarding the diagnosis of S*. mansoni*, however, zinc sulphate is a further supply to be considered, as it is more difficult to obtain and more expensive than saturated saline. It is to be taken into consideration that the two FS detect different helminths, as seen from the results we have shown above, FS2 is more sensitive than FS7 for hookworm (100% vs 86%), but does not detect *S.mansoni* whose eggs float only with FS7; this is due to the specific gravity of the solution that plays a crucial role in floatation of eggs. It is, therefore, particularly important to target the aim of the study and research before starting, for example, in settings where both STH and schistosomes are present, as around Lake Victoria, both floatation solutions are needed.

An additional advantage of the fill-FLOTAC and mini-FLOTAC system is that it is a “closed” method, in the sense that the stool and the solution are mixed in closed containers and the system is thoroughly safe with no risk of contamination for the operator. Formalin or any preservative can be added and the samples stored for further testing.

## Conclusion

To conclude, results suggest that mini-FLOTAC is a good and valid alternative to standardized methods, which can be easily transferred to resource-limited settings to monitor large scale treatment interventions for STH and to assess drug efficacy. Mini-FLOTAC, however, still needs to be compared with other quantitative techniques such as the McMaster method, and tested for faecal egg counts in settings where other helminth infections, other than hookworm and *S. mansoni*, are endemic.

## Competing interests

GC is the inventor and current patent holder of the Mini-FLOTAC apparatus. In case the currently on-going development and validation of the Mini-FLOTAC apparatus is successful, the method will be licensed free of charge to the World Health Organization and interested public non-commercial research centres. All other authors have no conflicts of interest.

## Authors’ contributions

BB, MA, LR and GC designed and coordinated the study and drafted the manuscript. BB, HZ carried out the parasitological analyses in Bukumbi. BB, LR performed the statistical analyses. MC, MA and RB contributed significantly to the revision of the manuscript. All authors read and approved the final version of the manuscript.
